# Weight gain attempts and diet modification efforts among adults in five countries: a cross-sectional study

**DOI:** 10.1186/s12937-022-00784-y

**Published:** 2022-05-13

**Authors:** Kyle T. Ganson, Jason M. Nagata, Lana Vanderlee, Rachel F. Rodgers, Jason M. Lavender, Vivienne M. Hazzard, Stuart B. Murray, Mitchell Cunningham, David Hammond

**Affiliations:** 1 Factor-Inwentash Faculty of Social Work, University of Toronto, Toronto, ON Canada; 2 Department of Pediatrics, Division of Adolescent and Young Adult Medicine, University of California, San Francisco, 550 16th Street., Box 0110, San Francisco, CA 94158 USA; 3 École de Nutrition, Centre de Nutrition, Santé Et Société (NUTRISS), Université Laval, Quebec City, QC Canada; 4 APPEAR, Department of Applied Psychology, Northeastern University, Boston, MA USA; 5 Department of Psychiatric Emergency & Acute Care, Lapeyronie Hospital, Montpellier, France; 6 Department of Medicine, Uniformed Services University, Bethesda, MD USA; 7 Military Cardiovascular Outcomes Research (MiCOR) Program, Bethesda, MD USA; 8 The Metis Foundation, San Antonio, TX USA; 9 Sanford Center for Bio-Behavioral Research, Sanford Health, Fargo, ND USA; 10 Department of Psychiatry and the Behavioral Sciences, University of Southern California, Los Angeles, CA USA; 11 School of Psychology, The University of Sydney, NSW Sydney, Australia; 12 School of Public Health and Health Systems, University of Waterloo, Waterloo, ON Canada

**Keywords:** Weight gain attempts, Diet modification, Food; diet, Calories, Muscularity

## Abstract

**Background:**

Recent research has emphasized a growing trend of weight gain attempts, particularly among adolescents and boys and young men. Little research has investigated these efforts among adults, as well as the specific diet modifications individuals who are trying to gain weight engage in. Therefore, the aims of this study were to characterize the diet modification efforts used by adults across five countries who reported engaging in weight gain attempts and to determine the associations between weight gain attempts and concerted diet modification efforts.

**Methods:**

Cross-sectional data from the 2018 and 2019 International Food Policy Study, including participants from Australia, Canada, Mexico, the United Kingdom, and the United States (*N* = 42,108), were analyzed. In reference to the past 12 months, participants reported on weight gain attempts and diet modification efforts related to increased consumption of calories, protein, fiber, fruits and vegetables, whole grains, dairy products, all meats, red meat only, fats, sugar/added sugar, salt/sodium, and processed foods. Unadjusted (chi-square tests) and adjusted (modified Poisson regressions) analyses were conducted to examine associations between weight gain attempts and diet modification efforts.

**Results:**

Weight gain attempts were significantly associated with higher likelihood of each of the 12 forms of diet modification efforts among male participants, and 10 of the diet modification efforts among female participants. Notably, this included higher likelihood of efforts to consume more calories (males: adjusted prevalence ratio [aPR] 3.25, 95% confidence interval [CI] 2.94–3.59; females: aPR 4.05, 95% CI 3.50–4.70) and fats (males: aPR 2.71, 95% CI 2.42–3.03; females: aPR 3.03, 95% CI 2.58–3.55).

**Conclusions:**

Overall, the patterns of association between weight gain attempts and diet modification efforts may be indicative of the phenomenon of muscularity-oriented eating behaviors. Findings further highlight the types of foods and nutrients adults from five countries may try to consume in attempts to gain weight.

## Background

Research in Canada, the United States (U.S.), and the United Kingdom (U.K.) has shown that targeted weight gain attempts are common among the general population, particularly in boys and young men. Among young adult men and women ages 17 to 32 years in Canada, the prevalence of weight gain attempts is 23% and 6%, respectively [1]. Among the U.S. population, nearly one third of adolescent boys (30%) and over one quarter of young adult men ages 18 to 26 years (27%) report concerted weight gain attempts, in stark contrast to only 6% of adolescent girls and 5% of young adult women ages 18 to 26 years reporting weight gain attempts [2, 3]. Similarly, among adolescents in the U.K., prevalence of weight gain attempts is higher among adolescent boys (13%) than girls (4%) [4]. Recent research, however, has underscored the relatively common occurrence of weight gain attempts among an international sample of adults [5], indicating the global relevance of such weight-change efforts.

Among the most commonly reported methods utilized to gain weight among both adolescents and young adults is the adoption of specific diets or modifying food intake. For example, among young adults in Canada reporting weight gain attempts, 72% of men and over 50% of women reported consuming a greater volume of protein, while roughly 20% of both men and women reported eating more fat and 20% of men and 15% of women reported eating more carbohydrates [1]. This compares to 7% of young adult men and 2% of young adult women in the U.S. reporting eating different foods than usual to gain weight [3]. Among adolescents in the U. S., roughly two thirds of both boys and girls reported changing their eating to enhance their muscle size or tone [6], while 4% of adolescent boys and 1% of adolescent girls reported dieting to gain weight [3]. These data highlight weight gain as a motivating factor for trying to alter to one’s diet and food intake. However, aside from the study by Minnick et al. [1], the types of diet modification efforts (i.e., efforts to consume more or less of a particular food) among individuals reporting weight gain attempts remains poorly characterized.

This study therefore aimed to address several gaps in the literature. First, to date, much of the research on weight gain attempts has focused on adolescents and young adults, with a dearth of knowledge on the nature of weight gain attempts among adults reflecting the broader lifespan. Second, while studies on weight gain attempts among the general population have been conducted in multiple high-income countries (e.g., Canada, U.S., U.K.), the methodologies of these studies have differed, limiting the ability to conduct meaningful cross-cultural comparisons. Furthermore, there is little information from middle-income countries, such as Mexico, where food environments and dietary patters may differ from high-income countries. Lastly, research has provided a broad overview of the behavioral and diet modification efforts utilized to gain weight; however, these studies often lack specificity and a nuanced assessment of precisely which unique efforts to change diet and food intake were undertaken. Indeed, individuals—particularly boys and men—who are attempting to gain weight often attend closely to their intake of calories and specific macro and micro nutrients [7, 8]; investigation of specific diet modification efforts is therefore warranted to provide a clearer understanding of such weight gain behaviors. This is specifically needed in order to evaluate diet modifications in comparison to dietary guidelines proposed across countries that often emphasize “healthful” eating (e.g., increased consumption of whole grains, fruits, and vegetables, decreased consumption of saturated fats and processed foods) [9–13]. Given these gaps, the aims of this study were, first, to describe the types of diet modification efforts most commonly reported among adults endorsing weight gain attempts from five countries, and second, to determine the associations between weight gain attempts and specific types of diet modification efforts.

## Methods

Data from two survey years (2018; 2019) of the International Food Policy Study (IFPS) were analyzed for the current study. IFPS is an annual repeated cross-sectional survey conducted in Australia, Canada, Mexico, the United Kingdom, and the United States. Participants were recruited via Nielsen Consumer Insights Global Panel and their partners’ panels. Email invitations with unique survey links were sent to a random sample of panelists within each country after targeting for demographic groups. Data were collected via web-based surveys with adults aged 18 years and older. Potential respondents were screened for eligibility, age, and sex quota requirements. Respondents provided informed consent and received remuneration in accordance with their panel’s typical incentive structure (e.g., points-based or monetary rewards, chances to win prizes). Surveys were conducted in English in Australia and the U.K.; Spanish in Mexico; English or French in Canada; and English or Spanish in the U.S. The study was reviewed and received ethics clearance through a University of Waterloo Research Ethics Committee (ORE#30,829). A full description of the study methods can be found elsewhere [14].

A total of 28,684 participants completed the 2018 survey and 29,290 participants completed the 2019 survey. Respondents were excluded for the following reasons: region was missing, ineligible or had an inadequate sample size (i.e., Canadian territories); invalid response to a data quality question; survey completion time under 15 min; and/or invalid responses to at least three of 20 open-ended measures (2018: *N* = 5,860; 2019: *N* = 8,322). The majority of missing data for both survey years was due to region missing or ineligible (2018: 81%; 2019: 87.0%). The final samples for the 2018 and 2019 survey years were 22,824 and 20,968, respectively. Responses from participants (*n* = 1,684) who were surveyed both years had their data retained from the 2018 survey year, resulting in a total sample of 42,108 unique participants.

### Measures

Weight gain attempts were assessed using the question, “During the past 12 months have you tried to… gain weight”. This measure aligns with prior research investigating weight gain attempts [1, 4, 5, 15].

Diet modification efforts were assessed using the question, “Have you made an effort to consume more or less of the following in the past year?” Categories included: calories, protein, fiber, fruits and vegetables, whole grains, dairy products, all meats, red meat (e.g., beef) only, fats, sugar/added sugar, salt/sodium, and processed foods. These categories largely align with eating behaviors and nutrient groups outlined in dietary guidelines across the five countries [9–13]. Response options for each category included, “consume more,” “consume less,” “no effort made,” and “don’t know.” For the purposes of this study, responses were dichotomized to 0 = “consume less; no effort made; don't know’” and 1 = “consume more”. Self-rated diet quality was assessed using the question, “In general, how healthy is your overall diet?” Potential response options included, “poor,” “fair,” “good,” “very good,” “excellent,” and “don’t know.”

Sociodemographics were assessed via self-report. Specifically, sex at birth was assessed using the question, “What sex were you assigned at birth, meaning on your original birth certificate?” Response options included “male” and female”. Race/ethnicity was categorized into “majority,” “minority,” and “not stated” groups, in line with census questions asked in each country. Education was categorized as “low”, “medium”, or “high” according to country-specific criteria of the highest level of formal education attained. These categorizations of race/ethnicity and education are consistent with prior IFPS research, and enable comparisons across countries while permitting IFPS country-specific data to be compared to national census estimates [16–18]. Body mass index (BMI) was calculated based on self-reported height and weight measurements according to each country’s measurement unit (e.g., pounds, feet and inches; kg/m^2^). BMI was categorized into four classes: ≤ 18.49 (“underweight”); ≥ 18.50 to ≤ 24.99; (“normal weight”); ≥ 25.00 to ≤ 29.99 (“overweight”); and ≥ 30.00 (“obesity”) based on Centers for Disease Control and Prevention guidelines [19].

### Statistical analysis

Descriptive statistics were calculated to provide an overview of the sample characteristics. Chi-square tests and independent samples *t-*tests were used to examine sex differences. Unadjusted prevalence of diet modification efforts by weight gain attempts and sex, and weight gain attempts and country, were estimated. Chi-square tests were used to examine diet modification efforts by sex and country. Unadjusted prevalence of weight gain attempts by diet quality was estimated with chi-square tests used to determine differences between diet quality rating. Multiple modified Poisson regression analyses with robust error variance [20] were conducted to estimate the associations (reported as prevalence ratios) between weight gain attempts (independent variable) and all 12 diet modification effort types (dependent variables) while adjusting for age, race/ethnicity, education, BMI category, country, and survey year. We tested for effect modification by sex and found statistically significant interactions for all diet modification efforts (*p*’s < 0.05). Therefore, regression analyses were conducted in the overall sample and also stratified by sex. This aligns with prior research showing differing prevalence of weight gain attempts among males and females [1, 3, 4, 15, 21]. All analyses included post-stratification sample weights that are constructed using a raking algorithm with population estimates from the census in each country based on age group, sex, region, ethnicity (except in Canada) and education (except in Mexico). Therefore, percentages reported are inclusive of sample weights and may not correspond with observed n’s. Analyses were conducted in 2022 using Stata 17.1.

## Results

Among the sample of 42,108 participants, 51.0% were female (Table 1). The mean age of the overall sample was 45.5 years, and 78.5% of participants identified with a majority racial or ethnic group within their country. Overall, 10.4% (*n* = 1,900) of male participants endorsed weight gain attempts over the past 12 months, compared to 5.4% (*n* = 1,082) of female participants.

**Table 1:** Characteristics of Male and Female Participants from the 2018 and 2019 International Food Policy Study (*N* = 42,108)

	Overall (*N* = 42,108)	Males *n* = 20,641	Females *n* = 21,467	*p*^a^
	M (SD) / n (%)	M (SD) / n (%)	M (SD) / n (%)	
Age	45.5 (16.8)	45.2 (16.8)	45.8 (16.7)	< .001
Race/ethnicity^b^				.374
Majority group	34,414 (78.5%)	16,855 (77.8%)	17,559 (79.3%)	
Minority group	7,199 (20.3%)	3,557 (21.0%)	3,642 (19.5)	
Not stated	495 (1.2%)	229 (2.2%)	266 (1.2)	
Education^c^				< .001
Low	11,875 (43.1%)	5,361 (40.9%)	6,514 (45.1%)	
Medium	11,360 (21.8%)	5,317 (21.4%)	6,043 (22.2%)	
High	18,730 (34.8%)	9,893 (37.3%)	8,837 (32.4%)	
Not stated	143 (0.4%)	70 (0.4%)	73 (0.4%)	
BMI category (kg/m^2^)				< .001
Underweight (< 18.5)	1,173 (3.0%)	382 (2.2%)	791 (3.7%)	
Normal weight (≥ 18.5 to < 25.0)	14,660 (33.9%)	6,640 (31.9%)	8,020 (35.8%)	
Overweight (≥ 25.0 to < 30.0)	12,048 (27.9%)	7,127 (33.0%)	4,921 (23.0%)	
Obesity (≥ 30.0)	14,227 (35.2%)	6,492 (32.9%)	7,735 (37.4%)	
Country				< .001
Canada	8,317 (19.7%)	4,053 (20.1%)	4,264 (19.3%)	
Australia	7,598 (18.1%)	3,721 (18.5%)	3,877 (17.8%)	
United Kingdom	9,267 (22.0%)	4,521 (22.0%)	4,746 (22.0%)	
United States	8,577 (20.4%)	4,039 (20.1%)	4,538 (20.6%)	
Mexico	8,349 (19.8%)	4,307 (19.3%)	4,042 (20.3%)	
Weight gain attempts, past 12 months	2,982 (7.9%)	1,900 (10.4%)	1,082 (5.4%)	< .001

*Note*: Frequencies represent observed counts which may not directly match weighted percentages. Percentages are weighted using sample weights

*M* Mean, *SD* Standard deviation, BMI Body mass index

^a^Differences between sexes were determined using chi-square tests and independent samples *t-*tests

^b^Race/ethnicity categories in each country as per census questions asked in each country: Australia: majority = only speaks English at home, minority = speaks a language besides English at home; Canada: majority = “White (European descent)”, minority = any other race/ethnicity; Mexico: majority = nonindigenous, minority = indigenous; United Kingdom: majority = “White”, minority = any other race/ethnicity; United States: majority = “White”, minority = any other race/ethnicity

^c^Education was categorized as “low” (i.e., completed secondary school or less), “medium” (i.e., some postsecondary qualifications), or “high” (i.e., university degree or higher) according to country-specific criteria of the highest level of formal education attained

**Fig. 1:**
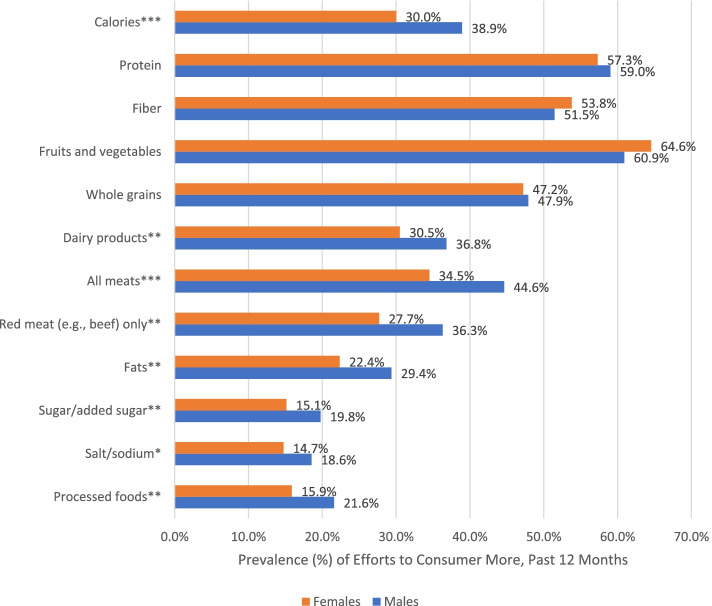
Prevalence of Diet Modification Efforts to Consume More in the Past 12 Months among Male and Female Participants who Reported Weight Gain Attempts from Five Countries in the 2018 and 2019 International Food Policy Study. Note: Chi-square tests for sex differences (*p < .05 **p < .01 ***p < .001). Analyses included sample weights

Unadjusted prevalence of diet modification efforts by sex among participants who reported weight gain attempts in the past 12 months are displayed in Fig. 1. Of the specific diet modification efforts assessed, efforts to consume more fruits and vegetables was most prevalent among both male and female participants who reported weight gain attempts (males: 60.9%; females: 64.6%), while efforts to consume more salt/sodium had the lowest prevalence (males: 18.6%; females: 14.7%). Significant sex differences emerged in the prevalence of diet modification efforts among male and female participants who reported weight gain attempts in the past 12 months, with all types of modifications reported more frequently by male versus female participants. This included attempts to consume more calories (males: 38.9%; females: 30.0%), dairy products (males: 36.8%; females: 30.5%), all meats (males: 44.6%; females: 34.5%), red meat only (males: 36.3%; females: 27.7%), fats (males: 29.4%; females: 22.4%), sugar/added sugar (males: 19.8%; females: 15.1%), and processed foods (males: 21.6%; females: 15.9%).

**Fig. 2:**
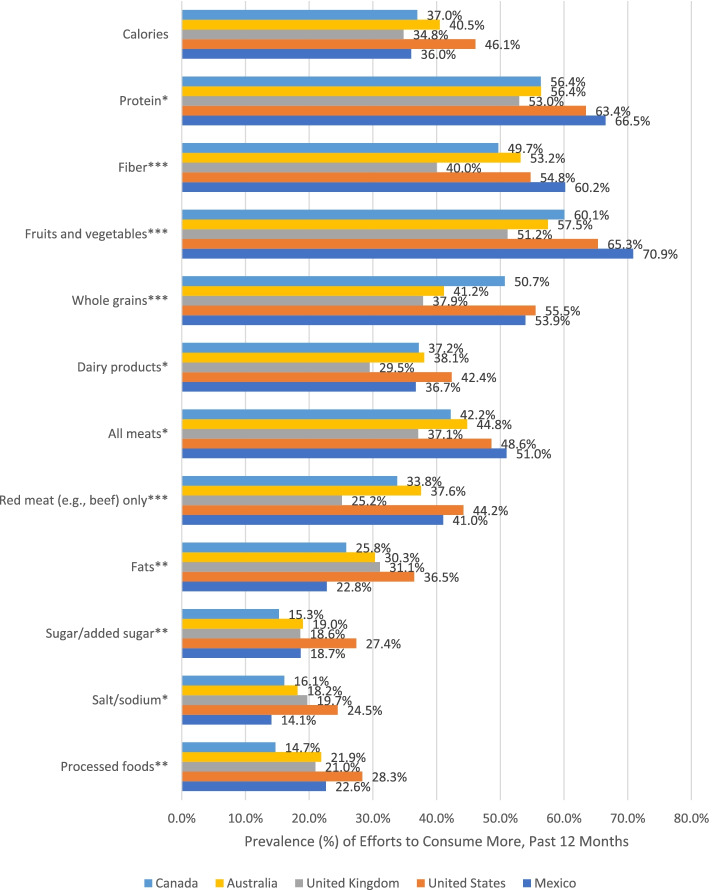
Prevalence of Diet Modification Efforts to Consume More in the Past 12 Months among Male Participants who Reported Weight Gain Attempts, by Country, in the 2018 and 2019 International Food Policy Study by Country. Note: Chi-square tests for country differences (*p < .05 **p < .01 ***p < .001). Analyses included sample weights

**Fig. 3:**
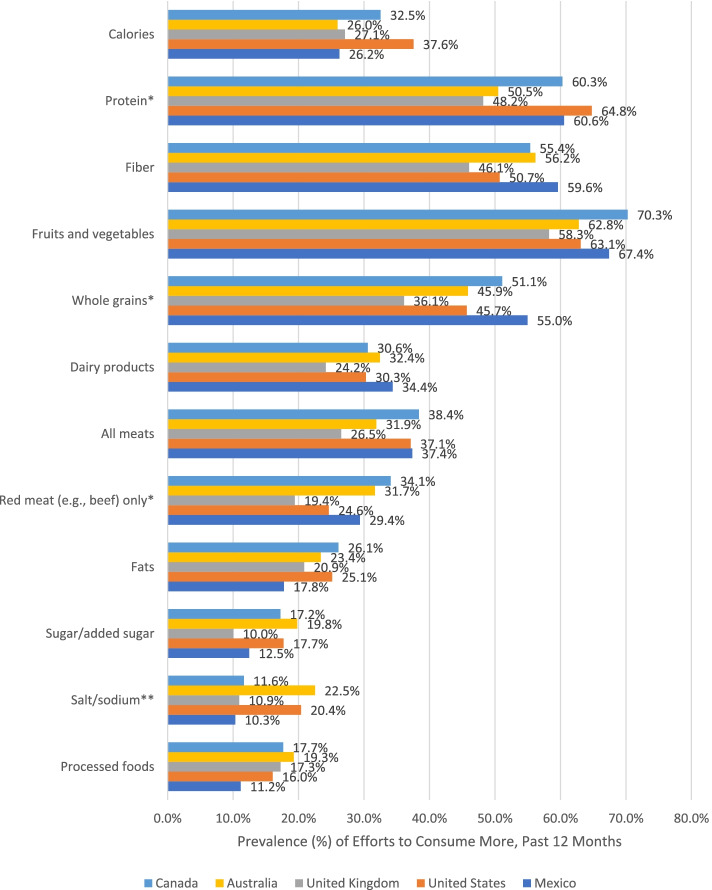
Prevalence of Diet Modification Efforts to Consume More in the Past 12 Months among Female Participants who Reported Weight Gain Attempts, by Country, in the 2018 and 2019 International Food Policy Study by Country. Note: Chi-square tests for country differences (*p < .05 **p < .01 ***p < .001). Analyses included sample weights

Unadjusted prevalence of several diet modification efforts differed significantly across the five countries. Among male participants who reported weight gain attempts in the past 12 months, efforts to consume more protein, dairy products, all meats, salt/sodium, fats, sugar/added sugar, processed foods, and fiber, fruits and vegetables, whole grains, and red meat only significantly differed across the five countries (Fig. 2). Among female participants who reported weight gain attempts in the past 12 months, efforts to consume more protein, whole grains, fats, and salt/sodium significantly differed across the five countries (Fig. 3).

**Fig. 4:**
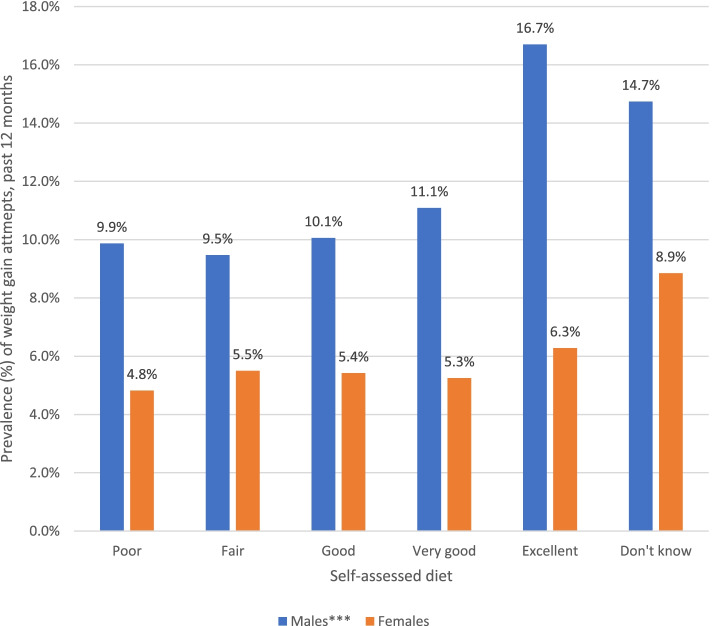
Prevalence of Weight Gain Attempts by Self-Rated Diet Quality among Male and Female Participants in the 2018 and 2019 International Food Policy Study. Note: Chi-square tests for differences in self-rated diet (***p < .001). Analyses included sample weights

The unadjusted prevalence of weight gain attempts by self-rated diet quality among male and female participants is displayed in Fig. 4. Among male participants, weight gain attempts were most common among participants who rated their diet as “excellent” (16.7%). There were no significant differences between weight gain attempts and diet quality among female participants.

Modified Poisson regression analyses revealed significant associations between weight gain attempts and diet modification efforts in the overall sample and when analyses were stratified by sex, while adjusting for potential confounders (Table 2). In the overall sample, weight gain attempts were significantly associated with higher likelihood of efforts to consume more of all 12 types of dietary categories, with efforts to consume more calories (adjusted prevalence ratio [aPR] 3.51, 95% confidence interval [CI] 3.23–3.81) and fats (aPR 2.83, 95% CI 2.58–3.10) having the strongest effect sizes. Among male participants, weight gain attempts were significantly associated with higher likelihood of efforts to consume more of all 12 types of dietary categories, with efforts to consume more calories (aPR 3.25, 95% CI 2.94–3.59) and fats (aPR 2.71, 95% CI 2.42–3.03) having the strongest effect sizes. Among female participants, weight gain attempts were significantly associated with higher likelihood of 10 diet modification efforts, with efforts to consume more calories (aPR 4.05, 95% CI 3.05–4.70), fats (aPR 3.03, 95% CI 2.58–3.55), and sugar/added sugar (aPR 2.71, 95% CI 2.18–3.36) having the strongest effect sizes.

**Table 2:** Associations between Weight Gain Attempts and Diet Modification Efforts in the Past 12 Months among Male and Female Participants from the 2018 and 2019 International Food Policy Study (*N* = 42,108)

	Overall (*N* = 42,108)		Males *n* = 20,641		Females *n* = 21,467	
	PR^a^ (95% CI)	*p*	PR^b^ (95% CI)	*p*	PR^b^ (95% CI)	*p*
Diet modification efforts						
Consume more:^c^						
Calories	**3.51 (3.23–3.81)**	** < .001**	**3.25 (2.94–3.59)**	** < .001**	**4.05 (3.50–4.70)**	** < .001**
Protein	**1.35 (1.29–1.40)**	** < .001**	**1.39 (1.32–1.47)**	** < .001**	**1.26 (1.17–1.35)**	** < .001**
Fiber	**1.15 (1.10–1.21)**	** < .001**	**1.19 (1.12–1.26)**	** < .001**	**1.10 (1.02–1.18)**	**.015**
Fruits and vegetables	**1.07 (1.03–1.11)**	** < .001**	**1.12 (1.07–1.17)**	** < .001**	1.01 (0.95–1.07)	.834
Whole grains	**1.17 (1.12–1.23)**	** < .001**	**1.23 (1.15–1.30)**	** < .001**	1.08 (0.99–1.17)	.071
Dairy products	**1.58 (1.47–1.70)**	** < .001**	**1.61 (1.48–1.75)**	** < .001**	**1.47 (1.30–1.66)**	** < .001**
All meats	**1.76 (1.65–1.87)**	** < .001**	**1.76 (1.64–1.89)**	** < .001**	**1.72 (1.54–1.92)**	** < .001**
Red meat (e.g., beef) only	**1.86 (1.73–2.00)**	** < .001**	**1.87 (1.71–2.03)**	** < .001**	**1.83 (1.60–2.08)**	** < .001**
Fats	**2.83 (2.58–3.10)**	** < .001**	**2.71 (2.42–3.03)**	** < .001**	**3.03 (2.58–3.55)**	** < .001**
Sugar/added sugar	**2.39 (2.12–2.69)**	** < .001**	**2.24 (1.95–2.58)**	** < .001**	**2.71 (2.18–3.36)**	** < .001**
Salt/sodium	**2.16 (1.92–2.43)**	** < .001**	**2.05 (1.78–2.36)**	** < .001**	**2.40 (1.94–2.96)**	** < .001**
Processed foods	**2.12 (1.91–2.38)**	** < .001**	**1.97 (1.74–2.23)**	** < .001**	**2.54 (2.06–3.15)**	** < .001**

*Note*: Analyses included sample weights

**Boldface** indicates statistical significance *p* < 0.05

*PR* = Prevalence ratio, *CI* = Confidence interval

^a^Adjusted for age, sex, race/ethnicity, education, body mass index category, country, and survey year

^b^Adjusted for age, race/ethnicity, education, body mass index category, country, and survey year

^c^Reference group for all diet modification efforts: Consume less; No effort made; “Don't know”

## Discussion

This study is the first to characterize the diet modification efforts among adults reporting weight gain attempts across five middle- and high-income countries. Broadly, descriptive analyses indicated that among adults who reported weight gain attempts, the most commonly reported diet modification efforts were to consume more fruits and vegetables, protein, fiber, and whole grains. This was the case for both men and women across all five countries. However, in regression analyses, efforts to consume more fruits and vegetables, protein, fiber, and whole grains had among the weakest effect sizes in the overall sample and for both males and females, including no significant relationship between weight gain attempts and efforts to consume more fruits and vegetables and whole grains among females. Thus, while these diet modification efforts were common in adults reporting weight gain attempts, they were also common in the full sample irrespective of weight gain attempts, rather than unique to those trying to gain weight. This may be in part due to overall nutrition guidance and education for the population that focuses on increased consumption of healthful foods such as fruits and vegetables, whole grains, and fiber [9–13]. In contrast, efforts to consume more calories were somewhat less common among adults who reported weight gain attempts yet had the strongest effect size in adjusted analyses, including over three-fold higher prevalence among men and four-fold higher prevalence among women who reported weight gain attempts. This was followed by efforts to consume more fats, with roughly three-fold higher prevalence among both males and females who reported weight gain attempts. These findings highlight the high-calorie and high-fat dietary intake efforts of participants reporting weight gain attempts despite existing data suggesting that intentional efforts to gain weight centrally implicate an upregulation in protein consumption [22] and a downregulation of foods that are less calorically dense and may have little benefit to increasing weight and muscularity (i.e., fruits and vegetables, whole grains, and fiber). This finding is in unique contrast to participants’ self-rated diet quality, where those who reported weight gain attempts, males in particular, were more likely to rate their diet as “excellent.” Taken together, these findings emphasize that both males and females engage in a vast array of diet modification efforts alongside attempts to gain weight, some of which may not support overall healthier dietary patterns, as suggested by governmental and public health guidance from all five countries [9–13], and may subsequently be damaging to their health, despite positive self-ratings of their diets.

The findings from this study may underscore muscularity-oriented eating behaviors, which largely encompass dietary practices (e.g., increased protein intake) that are intended to increase muscle-mass, muscularity, and tone, and decrease body fat [7, 22, 23]. These body characteristics align with the predominant ideal body for men [24–26] and are becoming more emblematic of the ideal body for women [27, 28]. Evidence of muscularity-oriented eating behaviors include, first, 39% higher prevalence of efforts to consume more protein, 61% higher prevalence of efforts to consume more dairy products, 76% higher prevalence of efforts to consume more of all meats, 87% higher prevalence of efforts to consume more red meat only, and nearly three-fold higher prevalence of efforts to consume more fats for males who reported weight gain attempts compared to males in the general population, with similar results among females. Second, these dietary efforts may be characteristics of high protein, high fat, and ketogenic diets [29], which are claimed to catalyze fat loss along with the maintenance, or even increase, of muscle mass [30]. Third, muscularity-oriented eating behaviors also include the consumption of dietary supplements, such as protein powders and bars that are marketed for those engaging in weight training, muscle-building, and athletic activities. These products are often considered processed foods, which may in part be driving the prevalence of reported efforts to consume more processed food in this population. Fourth, while it may seem counterintuitive that participants who reported weight gain attempts also reported efforts to consume more salt/sodium, there is evidence that salt/sodium is an important factor in post-workout recovery [31], including adequate sodium levels playing a role in ensuring sufficient blood volume to transport nutrients to muscles [32]. This may also align with efforts to consume more sugar/added sugar given that sugars can help with the ﻿muscle glycogen resynthesis process post exercise [33, 34]. Finally, the higher prevalence of efforts to consume more of all 12 dietary categories among men, and 10 among women, may be related to “cheat meals” or “cheat days,” and similarly, the “bulking” phase of “bulk” and “cut” cycles that are contextualized within a muscularity-oriented tradition. These behaviors consist of cyclical patterns of the consumption of a high quantity of calorie dense foods for a specific period of time before returning to restrictive/restrained diet practices with the intention of conferring the benefits for muscle enhancement [7, 22, 23, 35, 36]. Taken together, these findings may provide initial evidence of the dietary practices intended for muscularity, leanness, and tone among adults who report weight gain attempts.

Regarding country-specific differences in diet modification efforts, there are several findings worth highlighting. While efforts to increase caloric intake were commonly reported in all countries, the dietary approaches to increasing calorie content appeared to differ between countries. For example, men in the U.S. who reported weight gain attempts also reported significantly higher prevalence of efforts to consume more red meat, fats, sugar/added sugar, salt/sodium, and processed foods, all of which may increase risk of adverse health outcomes (e.g., cardiovascular disease) if consumed in excess [37–39]. This is contrasted with men in Mexico who reported weight gain attempts also exhibiting a higher prevalence of efforts to consume more protein, fiber, fruits and vegetables, and all meats, which may indicate the attempt to gain weight through increased intake of foods often considered as “healthier.” Second, among women, fewer explainable patterns across countries emerged signifying differences in prevalence of diet modification efforts among those who reported weight gain attempts. Future research is needed to further describe unique diet modification efforts among women who report weight gain attempts.

### Strengths and limitations

This study includes several strengths. First, the IFPS includes a large and international sample of adult participants representing diverse racial/ethnic and age groups. Second, this study analysed multiple survey years with two different participant cohorts, which provides greater assurance that the findings are not unique to one point in time and instead may represent a descriptive pattern of behavior. Lastly, our analysis included an array of specific diet modification efforts, providing more detailed insights in the specific form of dietary intake changes and their associations with weight gain attempts.

Despite these strengths, limitations should be noted. First, given the sampling method used, the findings do not provide nationally representative estimates. However, the data and analyses were weighted using preconstructed sample weights based on country-specific census data in an attempt to maximize external validity. Second, all responses are based on self-report, which may increase recall and social desirability bias. Third, the diet modification effort question did not specify the purpose of the effort; therefore, we are only able to theorize, based on the associations found, that these efforts for increased dietary intake may be motivated at least in part or for some by a desire to gain weight. Fourth, a single item was used to assess weight gain attempts, and no information was collected on the frequency or type of behaviors specifically engaged in for this purpose, or the motivations for weight gain. As such, interpretations of these behaviors in relation to specific motivations (e.g., increased muscularity) are speculative; however, a large proportion of men report wanting to enhance their muscularity [40] and engage in muscle-enhancing behaviors [3], which provides evidence for our interpretation of the data. Nevertheless, future research focused on the motivations for weight gain will be needed. Lastly, data were cross-sectional, thus limiting the ability to infer causal relationships between the variables examined.

## Conclusion

This study aimed to identify the diet modification efforts used among adults from five countries who report weight gain attempts. Results showed that both male and female adults who reported weight gain attempts had significantly higher likelihood of reporting efforts to modify their diet by consuming more calories, protein, fiber, dairy products, meats, fats, sugar, salt, and processed foods. These findings add to a growing literature on individuals who endorse attempts to gain weight and begin to describe the specific types of dietary intake behaviors those individuals seek to alter. Healthcare professionals should assess for weight gain attempts to provide appropriate clinical oversight and guidance and evaluate whether or not the dietary behaviors undertaken by these individuals may potentially undermine their health. Public health professionals should ensure that prevention and intervention programming aimed towards those attempting to gain weight consider the unique diet modification efforts reported in this study. This programming should be aimed at ensuring individuals are ascribing to balanced eating patterns and reducing the use of potentially detrimental dietary practices.

## Data Availability

The International Food Policy Study is available to researchers. Please visit http://foodpolicystudy.com/ for more information.
